# Incremental value of preoperative right ventricular function in predicting moderate to severe acute kidney injury after heart transplantation

**DOI:** 10.3389/fcvm.2022.931517

**Published:** 2022-08-09

**Authors:** Shuangshuang Zhu, Yanting Zhang, Weihua Qiao, Yixuan Wang, Yuji Xie, Xin Zhang, Chun Wu, Guohua Wang, Yuman Li, Nianguo Dong, Mingxing Xie, Li Zhang

**Affiliations:** ^1^Department of Ultrasound, Union Hospital, Tongji Medical College, Huazhong University of Science and Technology, Wuhan, China; ^2^Clinical Research Center for Medical Imaging in Hubei Province, Wuhan, China; ^3^Hubei Province Key Laboratory of Molecular Imaging, Wuhan, China; ^4^Department of Cardiovascular Surgery, Union Hospital, Tongji Medical College, Huazhong University of Science and Technology, Wuhan, China

**Keywords:** acute kidney injury, heart transplantation, right ventricular function, tricuspid annular plane systolic excursion, RV fractional area change

## Abstract

**Background:**

Acute kidney injury (AKI) commonly occurs after heart transplantation (HTx), but its association with preoperative right ventricular (RV) function remains unknown. Consequently, we aimed to determine the predictive value of preoperative RV function for moderate to severe AKI after HTx.

**Materials and methods:**

From 1 January 2016 to 31 December 2019, all the consecutive HTx recipients in our center were enrolled and analyzed for the occurrence of postoperative AKI staged by the Kidney Disease: Improving Global Outcomes (KDIGO) criteria. Conventional RV function parameters, including RV fractional area change (RVFAC) and tricuspid annular plane systolic excursion (TAPSE), were obtained. The primary endpoint was moderate to severe AKI (the KDIGO stage 2 or 3). The secondary endpoints included the impact of AKI on intensive care unit (ICU) mortality, in-hospital mortality, and 1-year mortality.

**Results:**

A total of 273 HTx recipients were included in the study. Postoperative AKI occurred in 209 (77%) patients, including 122 (45%) patients in stage 1 AKI, 49 (18%) patients in stage 2 AKI, and 38 (14%) patients in stage 3 AKI. Patients with higher AKI stage had lower baseline estimated glomerular filtration rate (eGFR), more frequent diabetes, higher right atrial pressure (RAP), longer cardiopulmonary bypass (CPB) duration, more perioperative red blood cell (RBC) transfusions, and worse preoperative RV function. A multivariate logistic regression model incorporating previous diabetes mellitus [odds ratio (OR): 2.21; 95% CI: 1.06–4.61; *P* = 0.035], baseline eGFR (OR: 0.99; 95% CI: 0.97–0.10; *P* = 0.037), RAP (OR: 1.05; 95% CI: 1.00–1.10; *P* = 0.041), perioperative RBC (OR: 1.18; 95% CI: 1.08–1.28; *P* < 0.001), and TAPSE (OR: 0.84; 95% CI: 0.79–0.91; *P* < 0.001) was established to diagnose moderate to severe AKI more accurately [the area under the curve (AUC) = 79.8%; Akaike information criterion: 274].

**Conclusion:**

Preoperative RV function parameters provide additional predicting value over clinical and hemodynamic parameters, which are imperative for risk stratification in patients with HTx at higher risk of AKI.

## Introduction

Heart transplantation (HTx) represents the mainstream therapy for end-stage heart disease, improving both their survival and quality of life (1, 2). Acute kidney injury (AKI) after HTx is a common complication in the early postoperative phase, which is associated with an unfavorable prognosis and may lead to subsequent progressive chronic kidney disease (3–9). A recent study revealed that right heart hemodynamic parameters obtained by right heart catheterization, including right atrial pressure (RAP) and pulmonary artery pulsatility index (PAPi), were strong predictors of the postoperative AKI after HTx (10). It is well-known that preexisting pulmonary hypertension increases right ventricular (RV) afterload, which can result in RV failure (11). Importantly, RV failure can shrink renal function by raising renal venous pressure, leading to congestive AKI (12, 13). However, the association of preoperative RV function with postoperative AKI early after HTx has not been established.

Due to the complex structure of the RV, cardiovascular MR (CMR) is considered the current reference standard for quantification of RV volume and function (14, 15). However, CMR is high cost and long image acquisition time are impediments to its widespread clinical use. In addition, some patients with end-stage heart failure before HTx cannot withstand long-term CMR examinations. Transthoracic echocardiography is a first-line, non-invasive method for evaluating RV function (1). Although tricuspid annular plane systolic excursion (TAPSE) and RV fractional area change (RVFAC) have been routinely assessed RV function, their value in predicting the occurrence of moderate to severe AKI after HTx remains unknown.

This study aimed to determine the predictive significance of routine RV function parameters measured by echocardiography at the time of transplantation listing in relation to moderate to severe AKI after HTx.

## Materials and methods

### Study population

We designed a retrospective study of consecutive adult patients who underwent HTx in the Wuhan Union Hospital, Tongji Medical College, Huazhong University of Science and Technology. We included all the adult patients transplanted between 1 January 2016 and 31 December 2019. Patients were excluded due to age < 18 years at the time of transplantation, underwent heart–lung transplantation, heart–kidney transplantation, retransplanted, renal replacement therapy (RRT) before transplantation, complex congenital heart disease, and poor image quality. Patient data were obtained from the hospital database, electronic medical records, and subsequent visit or contact.

This study was approved by the Ethics Committee of Wuhan Union Hospital, Tongji Medical College, Huazhong University of Science and Technology and was performed in accordance with the Declaration of Helsinki. All the donor grafts were donated to the Red Cross Society of Hubei Province and were allocated by the China Organ Transplant Response System. This study was in strict compliance with the International Society for Heart and Lung Transplantation (ISHLT) ethics statement. Furthermore, we obtained written informed consent from all the participants.

### Immunosuppressive protocol

Basiliximab and methylprednisolone were used for immune induction therapy. All the patients received methylprednisolone (20 mg/kg) intraoperatively when the aortic cross-clamp was released. Basiliximab (20 mg) was initiated in the operating room. The second dose (20 mg) was administered 4 days after HTx. This mediation was followed by a standard triple-drug immunosuppression regimen, including prednisone, calcineurin inhibitors, and mycophenolate mofetil. Prednisone started at an oral dose of 0.5 mg/kg/day and then tapered if the patient has no rejection events. Tacrolimus was the calcineurin inhibitor of the first choice with an oral dose of 0.10–0.15 mg/kg/day. Then, adjust the therapeutic dose of tacrolimus according to the concentration in the blood.

### Preoperative hemodynamic parameters

All the HTx candidates underwent right heart catheterization during the screening for transplantation listing unless they could not tolerate the examination. A balloon catheter was inserted through the femoral vein by using local anesthesia in the supine position. Procedural data were extracted from the catheterization reports and included the following parameters: RAP, pulmonary artery systolic pressure (PASP), pulmonary artery diastolic pressure (PADP), and PAPi [PAPi = (PASP − PADP)/RAP] (16–18).

### Echocardiography

Cardiac chamber sizes, and left ventricular (LV) and RV functions were measured based on the recommendations and guidelines of the American Society of Echocardiography (19). Comprehensive echocardiography, including M-mode, 2-dimensional, and color tissue Doppler modes, were obtained. LV internal diameter, and interventricular septal and posterior wall thicknesses were determined at end-diastole from the 2-dimensional mode of the parasternal long-axis. LV end-systolic and end-diastolic volumes, and LV ejection fraction (LVEF) were calculated with the biplane Simpson’s method. LV mass was calculated according to the Devereux formula (20). LV diastolic function was assessed by the ratio of early transmitral flow velocity (E) to early diastolic septal tissue velocity (e’) in the apical 4-chamber view. RV end-diastolic and end-systolic areas were measured from the apical 4-chamber view to calculate RV fractional area change (RVFAC). RVFAC was calculate as [(RV end-diastolic area − RV end-systolic area)/RV end-diastolic area] × 100%. Tricuspid annular peak systolic excursion (TAPSE) was obtained using M-mode echocardiography of the lateral annulus. Images were analyzed by 2 independent observers (SSZ and YTZ) blinded to clinical data.

### Study endpoints and definitions

Acute kidney injury was defined according to the Kidney Disease: Improving Global Outcomes (KDIGO) criteria (21). Baseline creatinine was defined as the most recent outpatient value before transplantation. If unavailable, the first creatinine values at hospital admission were accepted as the baseline. The baseline creatinine and highest postoperative creatinine value within the first week after HTx were used to classify the stage of AKI. AKI stage 1 was defined as serum creatinine increased by ≥0.3 mg/dl (≥26.5 μmol/l) or by 1.5–1.9 times from baseline, AKI stage 2 was defined as serum creatinine increased by 2.0–2.9 times from baseline, and AKI stage 3 was defined as serum creatinine increased by 3.0 times from baseline or by ≥4.0 mg/dl (≥ 353.6 μmol/l) or starting RRT (21). The primary endpoint was moderate to severe AKI (the KDIGO stage 2 or 3). The secondary endpoints included the impact of AKI on intensive care unit (ICU) mortality, in-hospital mortality, and 1-year mortality.

### Statistical analysis

All the statistical analyses were performed using the SPSS version 24.0 (SPSS Incorporation, Chicago, IL, United States) and R version 3.6.3 (R Foundation for Statistical Computing, Vienna, Austria).

Statistical charts were generated using Prism 7 (GraphPad software) and Minitab (version 18). A two-sided *p* < 0.05 was considered statistically significant. The linear trend across the AKI stage was performed by ANOVA or the Kruskal–Wallis test for continuous variables, and the chi-squared test was used for categorical variables. To determine the optimal cutoff value (maximum Youden index) of RV functional parameters for detecting the more severe forms of AKI (the KDIGO stage 2 or 3), the receiver operating characteristic (ROC) curves were used. Estimations of the predictors of postoperative AKI stages 2 and 3 were performed using the univariate and multivariate logistic regression models. Variables with *p*-values < 0.05 in univariate analysis were entered into multivariate logistic regression models. To assess the additional prognostic value of RV function parameters over other clinical variables, likelihood ratio tests were performed, and areas under the receiver operating characteristic curves (AUCs) and the Akaike information criterion (AIC) were calculated. Survival curves were obtained in the Kaplan–Meier analysis and compared using the log-rank test.

## Results

### Prevalence and trends of postoperative acute kidney injury

**FIGURE 1:**
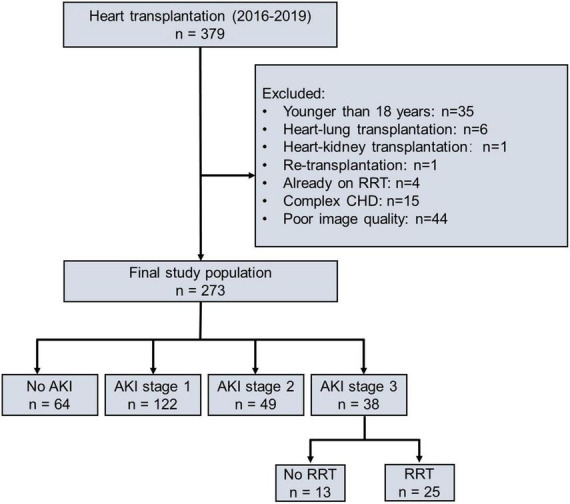
Flowchart of study population according to postoperative AKI severity. AKI, acute kidney injury; RRT, renal replacement therapy; CHD, complex congenital heart disease.

From 1 January 2016 to 31 December 2019, 379 patients underwent HTx at the Wuhan Union Hospital, of which 35 patients were under 18 years old, 6 patients underwent heart–lung transplantation, 1 patient underwent heart–kidney transplantation, 1 patient underwent retransplantation, 4 patients required RRT preliminary to transplantation, 15 patients had complex congenital heart disease, and 44 patients did not have images of sufficient quality, respectively. Finally, 273 patients were included in this study (Figure 1). Of 273 patients, 209 (77%) patients occurred AKI, including 122 (45%) patients in stage 1 AKI, 49 (18%) patients in stage 2 AKI, and 38 (14%) patients in stage 3 AKI. Of those who developed AKI stage 3, 25 (9%) patients required RRT which lasted for a median of 6 days [interquartile range (IQR) 3–14]. The incidence of AKI is given in Supplementary Figure 1. The time distribution for the occurrence of AKI with the highest peaks for all the three stages is shown in Supplementary Figure 2. Patients included in the study did not differ from those excluded with regard to recipient age, sex, body mass index (BMI), etiology of transplantation, baseline eGFR, or overall incidence of AKI (Supplementary Table 1).

### Demographic and perioperative data

Table 1 shows the baseline characteristics and perioperative data stratified by the AKI stage. Patients who had higher AKI stage shows lower baseline eGFR (88, 80, 77, and 64 ml/min/1.73 m^2^, *P* trend < 0.001), more frequent diabetes (11, 15, 20, and 26%, *P* trend = 0.028), higher baseline RAP (12, 11, 13, and 17 mm Hg, *P* trend < 0.001), longer cardiopulmonary bypass (CPB) duration (103, 110, 118, and 131, *P* trend < 0.001), and more perioperative red blood cell (RBC) transfusions (6, 7, 8, and 13, *P* trend < 0.001). Postoperatively, they were more likely to be diagnosed with RVF (6, 5, 18, and 39%, *P* trend < 0.001), primary graft failure (2, 2, 4, and 16%, *P* trend < 0.001), and the risk of rethoracotomy was higher (0, 0.8, 6, and 26%, *P* trend < 0.001). Therefore, patients with a higher AKI stage required more intra-aortic balloon pump (IABP) (30, 33, 53, and 58%, *P* trend < 0.001), and extracorporeal membrane oxygenation (ECMO) support (2, 0, 10, and 21%, *P* trend < 0.001).

**TABLE 1:** Baseline characteristics and perioperative data according to the postoperative AKI stage.

	No AKI, *n* = 64	AKI stage 1, *n* = 122	AKI stage 2, *n* = 49	AKI stage 3, *n* = 38	*P*-value
n (%)	64 (23)	122 (45)	49 (18)	38 (14)	
**Demographics**					
Age, years	47 ± 12	51 ± 11	49 ± 11	49 ± 11	0.637
Male sex	47 (73)	101 (83)	42 (86)	28 (74)	0.719
Weight, kg	62 ± 13	66 ± 12	68 ± 16	63 ± 11	0.517
Height, cm	168 ± 7	168 ± 7	168 ± 8	166 ± 8	0.113
BMI, kg/m^2^	21.7 ± 3.7	23.3 ± 3.7	23.9 ± 4.6	22.8 ± 3.4	0.096
**Primary cardiac disease**					0.084
Dilated cardiomyopathy	40 (62)	66 (54)	23 (47)	20 (53)	
Ischemic cardiac disease	15 (23)	25 (20)	12 (24)	7 (18)	
Others	9 (15)	31 (25)	14 (28)	11 (29)	
Duration of heart failure, years	5 (1–9)	5 (2–11)	7 (2–10)	5 (2–12)	0.185
**Renal function at baseline**					
eGFR, ml/min/1.73m2	88 ± 22	80 ± 22	77 ± 26	64 ± 19	< 0.001
eGFR ≥ 90	30 (47)	37 (30)	16 (33)	2 (5)	< 0.001
eGFR 60–89	29 (45)	65 (53)	21 (51)	22 (58)	
eGFR < 60	5 (8)	20 (16)	12 (24)	14 (37)	
eGFR 45–59	3 (5)	17 (14)	7 (14)	7 (18)	
eGFR < 45	2 (3)	3 (2)	5 (10)	7 (18)	
**Medical history**					
Diabetes mellitus	7 (11)	18 (15)	10 (20)	10 (26)	0.028
Hypertension	9 (14)	18 (15)	13 (26)	9 (24)	0.070
History of smoking	26 (41)	65 (53)	27 (55)	11 (29)	0.463
History of alcoholism	15 (23)	37 (30)	18 (37)	9 (24)	0.642
Prior cardiac surgery	7 (11)	14 (11)	4 (8)	5 (13)	0.888
Preoperative proteinuria	12 (19)	14 (11)	2 (4)	8 (21)	0.054
Preoperative albumin, g/L	39 ± 5	39 ± 5	39 ± 5	39 ± 4	0.609
Hypoalbuminemia	9 (14)	16 (15)	8 (10)	5 (13)	0.926
Preoperative hemoglobin, g/L	136 ± 17	134 ± 19	135 ± 20	131 ± 22	0.242
Anemia	8 (13)	24 (20)	8 (16)	10 (26)	0.151
**Donor characteristics**					
Age, years	35 ± 11	36 ± 11	35 ± 10	37 ± 12	0.413
Female sex	4 (6)	17 (14)	8 (16)	4 (10)	0.372
BMI, kg/m	23.0 ± 3.8	23.0 ± 2.7	22.2 ± 3.2	22.5 ± 3.8	0.391
Cause of death					0.714
Traumatic brain injury	37 (58)	74 (60)	28 (57)	21 (55)	
Cerebrovascular accident	19 (30)	36 (30)	18 (37)	15 (39)	
Other	8 (12)	12 (10)	3 (6)	2 (5)	
Time of ischemia donor heart, minutes	337 ± 82	339 ± 96	322 ± 94	321 ± 88	0.252
**Preoperative right heart catheterization parameters**					
Days before HTx	15 (8–27)	16 (8–27)	8 (3–24)	11 (5–19)	0.683
PASP, mmHg	47 ± 17	46 ± 16	47 ± 15	53 ± 17	0.056
PADP, mmHg	21 ± 9	19 ± 10	20 ± 8	24 ± 10	0.085
RAP, mmHg	12 ± 5	11 ± 6	13 ± 6	17 ± 8	< 0.001
PAPi	2.5 ± 1.1	2.6 ± 1.1	2.4 ± 1.1	2.1 ± 3.3	0.158
Perioperative (day 0 or 1) RBC, units	6 ± 3	7 ± 3	8 ± 4	13 ± 7	< 0.001
CPB duration, minutes	103 ± 27	110 ± 31	118 ± 44	131 ± 49	< 0.001
**Preoperative hemodynamic support**					
Inotropes	53 (83)	96 (79)	41 (84)	35 (92)	0.297
Vasopressors	41 (64)	76 (62)	33 (67)	33 (87)	0.041
Postoperative IABP	19 (30)	40 (33)	26 (53)	22 (58)	< 0.001
Postoperative ECMO	1 (2)	0 (0)	5 (10)	8 (21)	< 0.001
**Postoperative complications**					
Right ventricle failure	4 (6)	6 (5)	9 (18)	15 (39)	< 0.001
Primary graft failure	1 (2)	2 (2)	2 (4)	6 (16)	< 0.001
Re-thoracotomy	0 (0)	1 (0.8)	3 (6)	10 (26)	< 0.001

Values are mean ± SD, median (interquartile range) or number (percentage). AKI, acute kidney injury; BMI, body mass index; CPB, cardiopulmonary bypass; eGFR, estimated glomerular filtration rate; ECMO, extracorporeal membrane oxygenator; HTx, heart transplantation; IABP, intra-aortic balloon pump; RAP, right atrial pressure; RBC: red blood cell; PADP, pulmonary artery diastolic pressure; PASP, pulmonary artery systolic pressure; PAPi, pulmonary artery pulsatility index.

### Echocardiographic characteristics

**FIGURE 2:**
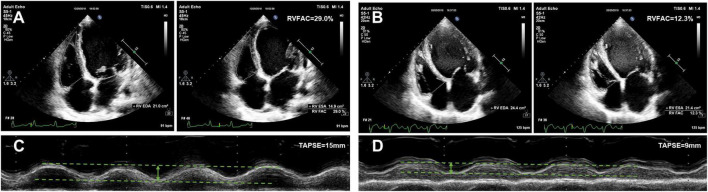
Representative examples of RVFAC and TAPSE measurement from patients awaiting heart transplant without AKI and with AKI stage 3. **(A)** RVFAC in the patient without AKI. **(B)** RVFAC in the patient with AKI stage 3. **(C)** TAPSE in the patient without AKI. **(D)** TAPSE in the patient with AKI stage 3. RVFAC, right ventricular fractional area change; TAPSE, tricuspid annular plane systolic excursion.

The echocardiographic characteristics according to the AKI stage are given in Table 2. The median time from echocardiographic examination to HTx was 17 days. Patients with a higher AKI stage displayed a larger RA dimension (45, 46, 46, and 55 mm, *P* trend < 0.001), and a worse RV function (TAPSE 17, 17, 15, and 12 mm, *P* trend < 0.001; RVFAC 28, 28, 26, and 22%, *P* trend < 0.001). Representative examples of RVFAC and TAPSE measurement from patients awaiting heart transplant without AKI and with AKI stage 3 are shown in Figure 2. However, left atrial, LV, RV, pulmonary artery (PA), and inferior vena cava (IVC) dimension, LV mass, E/e’ ratio, LV end-diastolic volume, LV end-systolic volume, LVEF, and moderate to severe tricuspid regurgitation did not differ among the AKI stages.

**TABLE 2:** Echocardiographic characteristics according to the postoperative AKI stage.

	No AKI, *n* = 64	AKI stage 1, *n* = 122	AKI stage 2, *n* = 49	AKI stage 3, *n* = 38	*P*-value
Days from echocardiographic examination to HTx	16 (8, 28)	18 (10, 32)	23 (11, 34)	16 (12, 24)	0.719
**Left heart**					
LA dimension, mm	53 ± 9	54 ± 10	54 ± 10	54 ± 9	0.857
LV dimension, mm	74 ± 13	76 ± 14	71 ± 13	70 ± 15	0.050
LV mass, g/m^2^	288 ± 114	323 ± 122	335 ± 172	276 ± 117	0.650
E/e’	29 ± 16	29 ± 16	25 ± 11	30 ± 10	0.808
LVEDV, mL	283 ± 128	292 ± 138	265 ± 127	234 ± 132	0.055
LVESV, mL	225 ± 120	228 ± 121	208 ± 111	185 ± 102	0.068
LVEF,%	22 ± 8	23 ± 9	23 ± 9	23 ± 10	0.585
**Right heart**					
RA dimension, mm	45 ± 7	46 ± 11	46 ± 10	55 ± 14	< 0.001
RVdimension, mm	44 ± 7	43 ± 9	42 ± 9	46 ± 11	0.199
PA (mm)	29 ± 5	29 ± 5	28 ± 5	30 ± 6	0.106
IVC (mm)	24 ± 6	25 ± 7	27 ± 6	26 ± 6	0.105
End-diastolic RV area, cm^2^	25 ± 9	23 ± 8	24 ± 9	28 ± 11	0.215
End-systolic RV area, cm^2^	18 ± 8	17 ± 7	18 ± 8	22 ± 10	0.013
RV-FAC, %	28 ± 8	28 ± 8	26 ± 9	22 ± 8	< 0.001
RV-FAC < 30%	41 (64)	72 (59)	33 (67)	34 (89)	0.009
TAPSE, mm	17 ± 4	17 ± 5	15 ± 4	12 ± 4	< 0.001
TAPSE < 17 mm	32 (50)	66 (54)	35 (72)	33 (87)	< 0.001
Moderate to severe TR	27 (42)	32 (26)	14 (29)	19 (50)	0.554

Values are mean ± SD, median (interquartile range) or number (percentage). AKI, acute kidney injury; LA, left atrial; LV, left ventricular; LVEDV, left ventricular end-diastolic volume; LVESV, left ventricular end-systolic volume; LVEF, left ventricular ejection fraction; MR, mitral regurgitation; RA, right atrial; RV, right ventricular; RVFAC, right ventricular fractional area change; TAPSE, tricuspid annular plane systolic excursion; TR, tricuspid regurgitation.

### Predictors of postoperative moderate to severe acute kidney injury

In Table 3, the univariate and multivariate logistic regression analyses were performed to find predictors of the more severe forms of AKI (the KDIGO stage 2 or 3). A univariate logistic regression analysis showed that diabetes mellitus [odds ratio (OR): 2.34; 95% CI: 1.25 to 4.39; *P* = 0.008], baseline eGFR (OR: 0.98; 95% CI: 0.97–0.99; *P* < 0.001), RAP (OR: 1.08; 95% CI: 1.03–1.12; *P* < 0.001), CPB duration (OR: 1.19; 95% CI: 1.07–1.32; *P* = 0.002), perioperative red blood cell (OR: 1.21; 95% CI: 1.13–1.31; *P* < 0.001), RVFAC (OR: 0.94; 95% CI: 0.91–0.97; *P* < 0.001), and TAPSE (OR: 0.82; 95% CI: 0.76–0.88; *P* < 0.001) were associated with moderate to severe AKI. However, recipient age, BMI, hypoalbuminemia, anemia, PASP, PAPi, donor age, gender and ischemia time of the donor’s heart, and LVEF were not predictive of moderate to severe AKI in univariate analysis. In multivariate logistic regression analysis, diabetes mellitus, baseline eGFR, RAP, perioperative red blood cell, TAPSE, and RVFAC continued to be of prognostic value.

**TABLE 3:** Predictors of the postoperative moderate to severe AKI by the univariate and multivariate logistic regression models.

	Univariate logistic regression		Multivariate logistic regression					
		
			Model 1		Model 2		Model 3	
	OR (95% CI)	*P*-value	OR (95% CI)	*P*-value	OR (95% CI)	*P*-value	OR (95% CI)	*P*-value
Recipient age, years	0.10 (0.97–1.02)	0.741						
Recipient BMI, kg/m^2^	1.05 (0.98–1.12)	0.166						
Previous Diabetes mellitus	2.34 (1.25–4.39)	0.008	2.08 (1.04–4.14)	0.038	2.17 (1.07–4.41)	0.033	2.21 (1.06–4.61)	0.035
Baseline eGFR, mL/min per 1.73 m^2^	0.98 (0.97–0.99)	< 0.001	0.98 (0.97–0.10)	0.014	0.98 (0.97–0.99)	0.005	0.99 (0.97–0.10)	0.037
Hypoalbuminemia	1.13 (0.55–2.34)	0.738						
Anemia	1.26 (0.66–2.39)	0.488						
PASP, mmHg	1.01 (0.99–1.03)	0.289						
RAP, mmHg	1.08 (1.03–1.12)	< 0.001	1.06 (1.02–1.11)	0.008	1.06 (1.01–1.11)	0.024	1.05 (1.00–1.10)	0.041
PAPi	1.08 (1.03–1.12)	0.117						
CPB duration, per 15 min longer	1.19 (1.07–1.32)	0.002		0.087		0.101		0.143
Perioperative RBC, units	1.21 (1.13–1.31)	< 0.001	1.18 (1.09–1.28)	< 0.001	1.19 (1.10–1.30)	< 0.001	1.18 (1.08–1.28)	< 0.001
Donor age, years	1.01 (0.98–1.03)	0.667						
Donor gender (female)	1.19 (0.56–2.56)	0.652						
Time of ischemia donor heart, minutes	1.00 (0.99–1.00)	0.148						
LVEF, %	1.00 (0.97–1.03)	0.991						
RV-FAC, %	0.94 (0.91–0.97)	< 0.001			0.94 (0.91–0.98)	0.001		
TAPSE, mm	0.82 (0.76–0.88)	< 0.001					0.84 (0.79–0.91)	< 0.001

OR, odds ratio; CI, confidence interval; AKI, acute kidney injury; BMI, body mass index; eGFR, estimated glomerular filtration rate; RAP, right atrial pressure; PAPi, pulmonary artery pulsatility index; PASP, pulmonary artery systolic pressure; RBC, red blood cell; TAPSE, tricuspid annular plane systolic excursion; RVFAC, right ventricular fractional area change.

**FIGURE 3:**
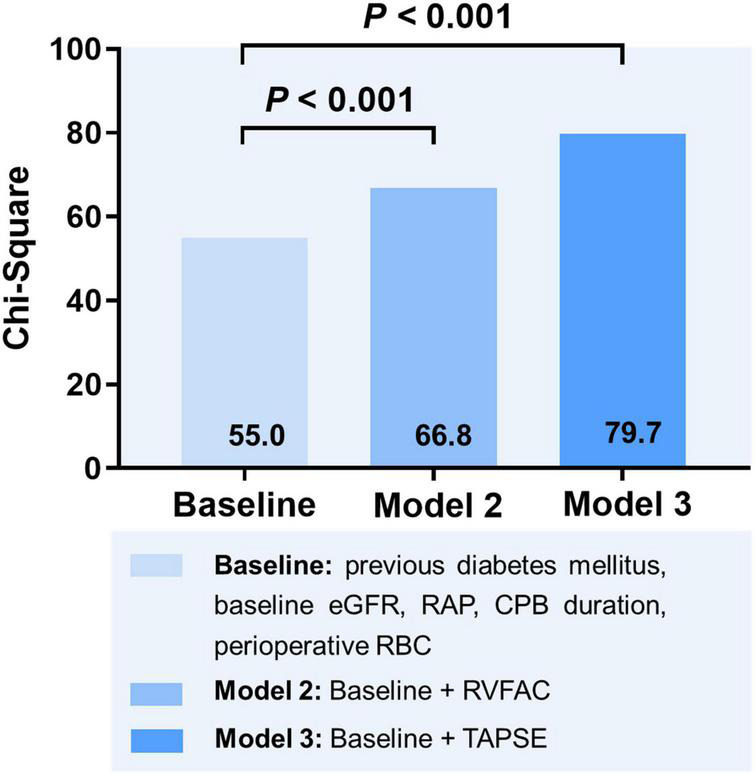
Likelihood ratio test for the incremental prognostic value of RV function parameters. eGFR, estimated glomerular filtration rate; RAP, right atrial pressure; CPB, cardiopulmonary bypass; RBC, red blood cell; RVFAC, right ventricular fractional area change; TAPSE, tricuspid annular plane systolic excursion.

**FIGURE 4:**
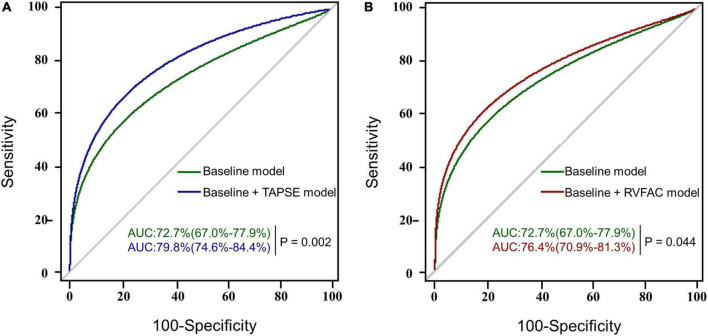
The AUC analysis for the prediction of postoperative moderate to severe AKI. RVFAC, right ventricular fractional area change; TAPSE, tricuspid annular plane systolic excursion; AUC, area under the receiver operating characteristic (ROC) curve with a *p*-value for the difference between different models. **(A)** The ROC curves of the baseline model (green) and baseline model + TAPSE (blue) for the prediction of moderate to severe AKI with the AUC and corresponding 95% confidence intervals. **(B)** The ROC curves of the baseline model (green) and baseline model + RVFAC (red) for the prediction of moderate to severe AKI.

**FIGURE 5:**
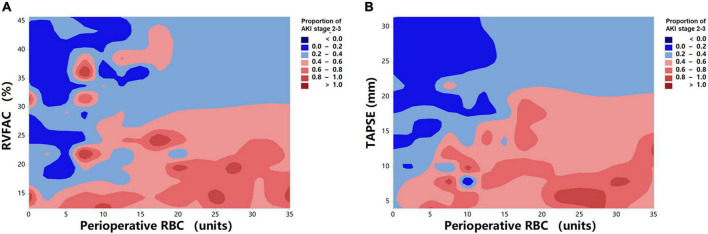
Contour plot of the risk of moderate to severe AKI in patients with HTx. Decreased RVFAC **(A)** and TAPSE **(B)** are associated with an increased risk of moderate to severe AKI, which is pronounced in patients with more perioperative RBC transfusions.

To determine the incremental value of RV function parameters in addition to clinical and hemodynamic parameters, a likelihood ratio test was performed. Figure 3 compares the additional chi-square statistic value of TAPSE and RVFAC to increase the predictive value for moderate to severe AKI. After the addition of RVFAC to the baseline model, an increase in the chi-square value was observed (chi-square difference = 11.8; *P* < 0.001). After the addition of TAPSE to the baseline model, an increased chi-square value was noted (chi-square difference = 24.7; *P* < 0.001). Moreover, the model with TAPSE (AUC = 79.8%; AIC = 274) was the best in predicting mortality compared with those with RVFAC (AUC = 76.4%; AIC = 287), and baseline model (AUC = 72.7%; AIC = 298) (Figure 4). We performed a contour plot to determine the relationship between RV function parameters, perioperative red blood cells, and postoperative moderate to severe AKI. The results revealed that decreased RVFAC and TAPSE were associated with an increased risk of moderate to severe AKI, which was pronounced in patients with more perioperative RBC transfusions (Figure 5). According to Youden’s index, the optimal cutoff points for predicting moderate to severe AKI were TAPSE < 14 mm and RVFAC < 20%.

### Relationship of acute kidney injury with posttransplant duration of hospitalization and short-term outcome

**FIGURE 6:**
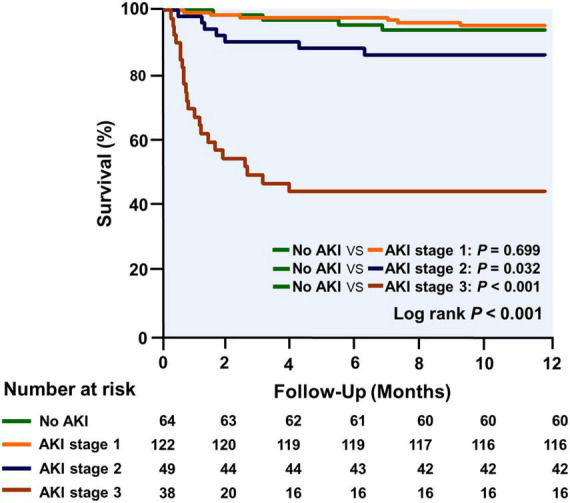
The Kaplan–Meier curves for survival analysis are stratified by AKI stage during the first year after transplantation.

Table 4 shows the effects of AKI severity on the duration of mechanical ventilation, ICU length of stay, and hospital stay after HTx. Patients who had a higher AKI stage had a longer mechanical ventilation (1,755, 2,095, 2,460, and 4,293 min, *P* trend < 0.001), ICU stay (5, 6, 8, and 13 days, *P* trend = 0.003), and hospital stay after HTx (36, 37, 47, and 54 days, *P* trend = 0.043). A trend was also seen in higher in ICU mortality (0, 0, 6, and 42%, *P* trend < 0.001) and hospital mortality with higher AKI stage (3, 2, 8, and 53%, *P* trend < 0.001). The overall 1-year mortality was 14.3% and the Kaplan–Meier survival curves stratified by AKI stage, and cumulative mortality of 6, 5, 14, and 58% for those without AKI and with AKI stages 1, 2, and 3, respectively (log-rank test, *P* trend < 0.001; Figure 6). There is no significant difference in survival between those without AKI stage 1 and with AKI stage 1. Patients with AKI stages 2 and 3 had a tendency to have worse short-term survival.

**TABLE 4:** The effects of AKI on the posttransplant duration of mechanical ventilation, hospitalization, and short-term outcome.

	No AKI	AKI stage 1	AKI stage 2	AKI stage 3	*P*-value
Duration of mechanical ventilation (min)	1755 (1313, 2742)	2095 (1410, 2570)	2460 (1600, 5450)	4293 (2625, 13260)	<0.001
Days in ICU	5 (4, 7)	6 (5, 8)	8 (6, 12)	13 (8, 21)	0.003
Days in hospital	36 (30, 47)	37 (29, 48)	47 (34, 57)	54 (33, 65)	0.043
**Short-term outcome**					
ICU mortality	0 (0)	0 (0)	3 (6)	16 (42)	<0.001
In-hospital mortality	2 (3)	2 (2)	4 (8)	20 (53)	<0.001
1-year mortality	4 (6)	6 (5)	7 (14)	22 (58)	<0.001

Values are median (interquartile range) or number (percentage). AKI, acute kidney injury; ICU, intensive care unit.

## Discussion

In this large, contemporary, real-world study based on retrospective clinical databases, we evaluated the incidence, predictors, and outcomes of AKI in patients undergoing HTx. The major findings of the present study are as follows: (i) AKI is a highly frequent complication in the early stage after HTx, and moderate to severe AKI (AKI stages 2 or 3) is associated with longer duration of mechanical ventilation, hospitalization, and worse short-term outcome; (ii) preoperative RV function is independently associated with moderate to severe AKI after HTx; (iii) more importantly, RV function parameters could provide additional predictive value over clinical and hemodynamic risk factors for moderate to severe AKI after HTx.

It is well-known that AKI is a common complication after HTx with an incidence ranging from 14 to 76%, which is associated with high morbidity and mortality in HTx recipients (3–9). As in previous studies, several independent risk factors for AKI after cardiac surgery were found, such as a history of diabetes, anemia, preoperative kidney function, RBC transfusions, and CPB duration (22–25). However, little is known about the impact of these factors on patients with HTx. Our results illustrate strong evidence of the relationship between pre- and perioperative risk factors and moderate to severe AKI after HTx. Previous diabetes mellitus, baseline eGFR, and perioperative RBC transfusions were independent predictors for the more severe forms of AKI.

It is known that preexisting pulmonary hypertension increases RV afterload, which can lead to RV dysfunction (11). Consequently, the right heart hemodynamic parameters have been routinely assessed in all the HTx candidates. Recently, increasing attention paid to the impact of the right heart hemodynamics on the development of AKI in various clinical settings, including HTx recipients (10, 26–32). The present study confirmed the predictive value of preoperative RAP for postoperative moderate to severe AKI. These results were in keeping with the findings of Guven et al., which demonstrated that preoperative RAP strongly predicted the development of AKI early after HTx after adjustment for known clinical risk factors (10). This may be explained by that long-standing venous congestion makes the kidneys more vulnerable to the development of AKI after HTx. The association between RV dysfunction and kidney injury was identified in several diseases (33–37). Wiersema et al. found that lower TAPSE was independently significantly associated with the development of AKI in critically ill patients (33). Mukherjee et al. showed that RV dysfunction was associated with kidney injury in patients with heart failure (36). Guinot et al. reported that patients with RV dysfunction had a higher risk for the development of renal dysfunction after non-HTx cardiac surgery (37). The link of preoperative RV function with AKI after HTx needs to be verified in HTx recipients.

Accordingly, our study revealed the important clinical implication of TAPSE and FAC, as they can be easily obtained during bedside echocardiography. Moreover, TAPSE appears to be a more robust predictor of AKI after HTx compared with RVFAC. Considering that RVFAC depends on RV endocardial definition, resulting in relatively poor reproducibility in subjects with suboptimal image quality. In contrast, TAPSE is less dependent on image quality and does not require geometric assumptions or RV endocardial definition, with high reproducibility and feasibility. To the best of our knowledge, this may be the first study to describe the RV function in relation to the occurrence of postoperative moderate to severe AKI after HTx.

## Limitations

Several limitations of our analyses should be noted. First, the main limitation of our study is a single-center retrospective study with relatively limited sample size. Future studies with multicenter and larger sample sizes are needed to determine the prognosis value of RV function in patients with HTx with AKI. Second, 44 patients had to be excluded from the study due to unavailable RV function data. However, when baseline characteristics were compared between patients who were included and excluded, we found no significant differences in these characteristics. In addition, TAPSE is load-dependent. RV longitudinal strain derived from 2-dimensional speckle-tracking echocardiography is less load-dependent, and its predictive value for AKI needs further investigation in the future. Lastly, we reported that the more severe forms of AKI were associated with ICU, in-hospital mortality, and 1-year mortality, but our database did not incorporate data on longer-term renal function or outcomes. To the best of our knowledge, the present study may be the largest-scale single-center cohort in China to report clinical incidence, predictors, and outcomes of postoperative AKI in HTx candidates.

## Conclusion

Our study shows that preoperative RV function parameters powerfully predict the development of moderate to severe AKI after HTx, offering incremental prognostic value over the clinical and hemodynamic parameters, which are important for risk stratification in patients with HTx at higher risk of AKI.

## Data availability statement

The raw data supporting the conclusions of this article will be made available by the authors, without undue reservation.

## Ethics statement

This study was approved by the Ethics Committee of Wuhan Union Hospital, Tongji Medical College, Huazhong University of Science and Technology and was performed in accordance with the Declaration of Helsinki. The patients/participants provided their written informed consent to participate in this study. Written informed consent was obtained from the individual(s) for the publication of any potentially identifiable images or data included in this article.

## Author contributions

SZ, YZ, WQ, ND, YL, MX, and LZ: conception and design of the study. SZ, YZ, WQ, YW, YX, XZ, and GW: acquisition of data. SZ, YZ, WQ, and CW: analysis and interpretation of data. SZ, WQ, and YZ: drafting the manuscript. SZ, ND, YL, MX, and LZ: final approval of the manuscript. All authors contributed to the article and approved the submitted version.

## Supplementary material

Supplementary Figure 1 The proportion of postoperative AKI according to severity stage during the period from 2016 to 2019. The X-axis depicts 1-year intervals. The Y-axis depicts the proportion of patients per each 1-year category according to the AKI stage. AKI, acute kidney injury.

Supplementary Figure 2 Time distribution of AKI occurrence according to the severity stage during the first 7 postoperative days. The X-axis depicts the time in days; The Y-axis depicts the proportion of patients per AKI stage. AKI, acute kidney injury; HTx, heart transplantation.
