# Supplementary figures and images for: Incremental value of preoperative right ventricular function in predicting moderate to severe acute kidney injury after heart transplantation
Source: Front Cardiovasc Med. 2022 Aug 9;9:931517. doi: 10.3389/fcvm.2022.931517 (PMC9398196; doi:10.3389/fcvm.2022.931517)

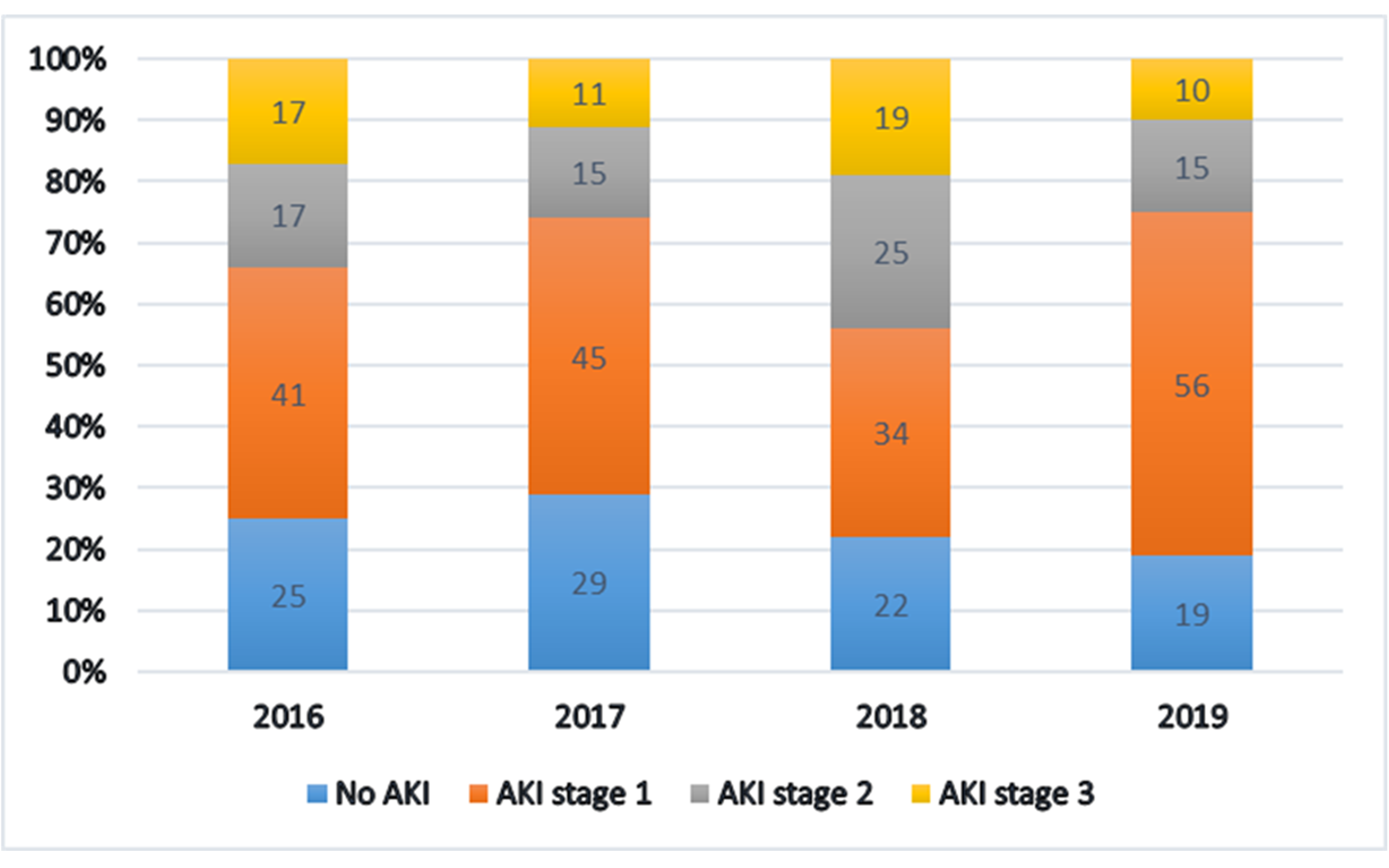

Supplement: Supplementary Figure 1 — The proportion of postoperative AKI according to severity stage during the period from 2016 to 2019. The X-axis depicts 1-year intervals. The Y-axis depicts the proportion of patients per each 1-year category according to the AKI stage. AKI, acute kidney injury. [file Image_1.TIF]

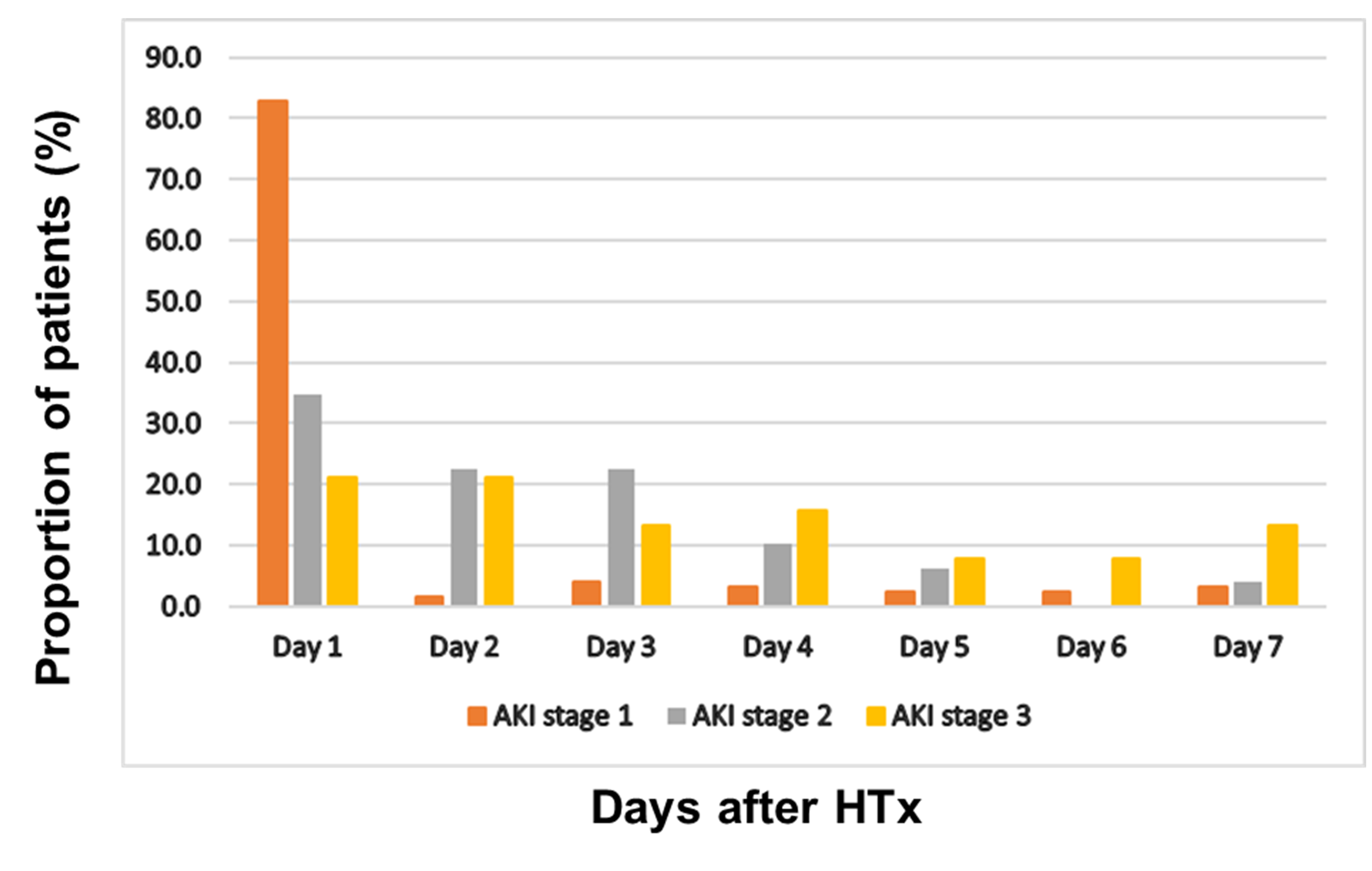

Supplement: Supplementary Figure 2 — Time distribution of AKI occurrence according to the severity stage during the first 7 postoperative days. The X-axis depicts the time in days; The Y-axis depicts the proportion of patients per AKI stage. AKI, acute kidney injury; HTx, heart transplantation. [file Image_2.TIF]
