# Supplementary material for: Incremental value of preoperative right ventricular function in predicting moderate to severe acute kidney injury after heart transplantation
Source: Front Cardiovasc Med. 2022 Aug 9;9:931517. doi: 10.3389/fcvm.2022.931517 (PMC9398196; doi:10.3389/fcvm.2022.931517)
Supplement: Supplementary file 3 [file Table_1.DOCX]

| **Supplementary Table 1. Baseline Characteristics of patients who were included and excluded** | | | |
| --- | --- | --- | --- |
|  | Included (n=273) | Excluded (n=44) | *P* value |
| No AKI (n, %) | 64 (23) | 10 (23) | 0.981 |
| AKI stage 1 (n, %) | 122 (45) | 21 (48) |  |
| AKI stage 2 (n, %) | 49 (18) | 7 (16) |  |
| AKI stage 3 (n, %) | 38 (14) | 6 (14) |  |
| Demographics |  |  |  |
| Age, years | 49±11 | 47±13 | 0.328 |
| Male sex | 47 (73) | 47 (73) |  |
| BMI, kg/m^2^ | 22.9±3.9 | 23.6±4.7 | 0.284 |
| Primary cardiac disease |  |  | 0.208 |
| Dilated cardiomyopathy | 149 (55) | 20 (46) |  |
| Ischemic cardiac disease | 59 (21) | 8 (18) |  |
| Others | 65 (24) | 16 (36) |  |
| Renal function at baseline |  |  |  |
| eGFR, ml/min/1.73m2 | 79±23 | 78±30 | 0.832 |
| eGFR ≥ 90 | 84 (31) | 15 (34) | 0.141 |
| eGFR 60-89 | 138 (50) | 16 (36) |  |
| eGFR < 60 | 51 (19) | 13 (30) |  |

Values are mean ± SD or number (percentage). AKI, acute kidney injury; BMI, body mass index; eGFR, estimated glomerular filtration rate.
